# Switching Between Second-Generation Antipsychotics in Patients with Schizophrenia and Schizoaffective Disorder: 10-Year Cohort Study in Brazil

**DOI:** 10.3389/fphar.2021.638001

**Published:** 2021-05-31

**Authors:** Izabela Fulone, Marcus Tolentino Silva, Luciane Cruz Lopes

**Affiliations:** Pharmaceutical Sciences Graduate Course, University of Sorocaba, Sorocaba, Brazil

**Keywords:** antipsychotics, agents, schizophrenia, schizoaffective disorder, drug substitution, Brazil

## Abstract

**Objective:** Switching between second-generation antipsychotics (SGAs) is a common clinical practice in the treatment of schizophrenia and schizoaffective disorders due to differences in the drugs’ tolerability and safety profiles as well as the challenge of obtaining an ideal response. However, the factors associated with SGA switching remain uncertain and related real-world data are scarce. The main objective was to identify the factors associated with the switching of SGAs in patients with schizophrenia or schizoaffective disorder.

**Methods:** We conducted a retrospective cohort study of outpatients with schizophrenia or schizoaffective disorder, who were aged ≥18 years and received a SGA (clozapine, olanzapine, risperidone, quetiapine or ziprasidone) from a Brazilian pharmaceutical assistance program for at least 3 months. We identified SGA users from 2008 to 2017 by using a national administrative database (Ambulatory Information System-SIA/SUS). The factors associated with the switches were evaluated by Cox proportional hazards regression and adjusted for sex and age; the confidence interval was set at 95% (95% CI).

**Results:** In total, 563,765 patients were included. Female sex, advanced age of ≥70 years, residence in the Brazilian northeast region, and the type of antipsychotic used were associated with an increased risk of switching (*p* < 0.001). The incidence of switching ranged from 37.6/100 person-years for clozapine users to 58.2/100 person-years for risperidone users. Compared to the adjusted hazard ratio, for clozapine users, the corresponding ratios for risperidone, ziprasidone, quetiapine and olanzapine were 1.59 (95% CI, 1.57–1.61), 1.41 (95% CI, 1.39–1.44), 1.25 (95% CI, 1.23–1.26) and 1.11 (95% CI, 1.10–1.12) respectively.

**Conclusion:** The groups most susceptible to SGA switching in real-life setting were older individuals, women, and those living in the Brazilian northeast region. Risperidone was associated with the highest risk of switching and as expected, clozapine was associated with the lowest risk of switching than that associated with the other SGAs.

## Introduction

Switching of antipsychotics in the treatment of schizophrenia or related psychotic disorders is a common clinical practice and has been recommended to ensure a more appropriate treatment option in the face of issues including efficacy, safety, or tolerability (Faries et al., 2009; Nyhuis et al., 2010). Therapeutic failure, including suboptimal improvement, persistence of negative or positive symptoms, worsening of certain symptoms or level of functioning, poor treatment adherence, and intolerable adverse reactions may determine the switch during treatment (Keks et al., 2019). The most common reasons for switching related to safety and tolerability, depending on the antipsychotic used, include weight gain, metabolic syndrome, sedation, hyperprolactinemia, anticholinergic effects, increased QT, and sexual dysfunction (Stroup and Gray, 2018)

The decision to switch from one antipsychotic to another should be made considering the possible risks associated with the switching process, such as relapses, exacerbation of some symptoms, new adverse effects, withdrawal syndromes, rebound, and serious drug interactions (Bernardo et al., 2011). Patients who switched antipsychotics were more likely to be hospitalized and use acute care services compared to patients continuing with the initial antipsychotic. In addition, switching resulted in poor clinical outcomes and higher total health care costs (Faries et al., 2009).

Antipsychotics switching could be influenced by various factors: in particular, factors related to the patient, illness, environment, antipsychotics availability, acceptability of treatment, and especially related to the medication (e.g., adverse events, drug interactions, therapeutic response) need to be evaluated (Buckley and Correll, 2008). First-generation and second-generation antipsychotics (SGAs) constitute heterogeneous groups of drugs with different receptor binding and affinity profiles, and hence, distinct clinical effects are expected (Cerovecki et al., 2013). Knowledge about the differences in the pharmacodynamic and pharmacokinetic characteristics of the previous and newer antipsychotics is crucial for adopting a more effective switching strategy, discontinuation strategy (abrupt discontinuation or tapering) for each agent, and optimization of the outcomes (Correll, 2010).

In case of an abrupt switch from an antipsychotic with high cholinergic affinity, such as olanzapine or clozapine, to that with a low affinity, cholinergic rebound syndrome is likely (Cerovecki et al., 2013). Switching from antipsychotics with high affinity for histaminergic receptors to those with low affinity will result in rebound insomnia (Buckley and Correll, 2008). Similarly, switching from clozapine to any other antipsychotic, can lead to serious withdrawal effects and rebound psychosis (Ganguli, 2002; Keks et al., 2019).

SGAs are considered high-cost medicines and are dispensed and funded by the Brazilian National Health System (SUS) after an analysis of compliance with the National Clinical Guidelines (Brazil, 2013; Brazil, 2014a). The costs of the treatment of schizophrenia are high worldwide, particularly pharmacological therapy (Santos et al., 2017). SGAs are responsible for the high cost of treatment for schizophrenia through SUS (Lindner et al., 2009). Patients refractory to other antipsychotics tend to undergo antipsychotics switching and have psychotic outbreaks followed by hospitalizations, which result in a significant economic burden in Brazil (Barbosa et al., 2018).

However, despite the burden and risks, few studies have explored the factors associated with switching antipsychotics in the treatment of schizophrenia and other psychotic disorders (Nyhuis et al., 2010; Xu and Krishnaswamy, 2018). Therefore, the aim of this study was to investigate the factors associated with switching of SGAs in patients with schizophrenia or schizoaffective disorder in Brazil.

## Methods

This study followed the REporting of studies Conducted using Observational Routinely collected health Data (RECORD) Statement which reporting items specific to observational studies using routinely collected health data (Benchimol et al., 2015).

### Study Design

This was a retrospective cohort study conducted in all patients who were on SGAs for schizophrenia or schizoaffective disorder treatment and received these medicines through a pharmaceutical assistance program from the SUS, from January 2008 to December 2017.

### Setting

The cohort was conducted using register-based data from a nationwide administrative database (Ambulatory Information System, SIA/SUS), where in information regarding all outpatient care procedures is collected, and processed and which is used for providing high-cost medicines or procedures for specific diseases according to Brazilian guidelines. This database represents over 200 million procedures/month (Brazil, 2008). These individualized secondary data are publicly available (unrestricted access) and were available from 2008.

The public health system in Brazil, SUS, is universal and free for everyone, and assists approximately 75.6% of the population (Brazil, 2014b). The private health system (health plan or direct payment) acts in a complementary way. Regardless of whether the medical prescription is from a private or public sector, the patient has the right to have access to medicines of high unitary value (e.g., SGA) via SUS, since the coverage of health policy is universal. As they are high-cost medicines, this can inflate the family budget and it is likely that some patients are receiving their pharmacotherapy via the SUS and using the private sector for outpatient and hospital treatment, since health plans in Brazil usually do not provide medicines such as SGA.

The patients get access to these medicines via SUS only after an analysis of compliance with Brazilian guidelines for the treatment of schizophrenia and schizoaffective disorders. So, patients with schizophrenia or schizoaffective disorder who seek to obtain these medicines fully funded by SUS should schedule a doctor’s appointment (from the public or private sector), which will make a proper diagnosis and then will fill out a form to request these medications. This form together with supporting documents are analyzed by the manager of pharmaceutical services and if everything is in accordance with the current Brazilian clinical protocol, the medicine is provided. This process guarantees the supply of medicines up to three months (90 days). After 90 days, if there is a need to continue treatment, the whole process is repeated. For this reason, we adopted the 90-days cut-off. All of this information such as sex, age, race, state of residence, diagnosis, prescribed SGA, dose, quantity dispensed by pharmacy, and date of dispensation are available for consultation and analysis at SIA/SUS database.

Currently, the following SGAs are available through the SUS: oral clozapine, olanzapine, quetiapine, risperidone, and ziprasidone. Aripiprazole or injectable SGAs are not available through the SUS.

Brazilian guidelines for schizophrenia or schizoaffective disorders treatment recommend SGA monotherapy (Brazil, 2013). There is no order of preference to use SGA, except clozapine. Clozapine is only used in cases in which patients are refractory to at least two other antipsychotics. Both Brazilian guidelines do not make specific recommendations for the elderly.

### Participants

The study included all patients aged ≥18 years, who were identified from SIA/SUS database, received SGAs (clozapine, olanzapine, quetiapine, risperidone, or ziprasidone) for more than 90 days, during January 2008 to December 2017, and diagnosed with one of the following diseases according to International Statistical Classification of Diseases and Related Health Problems, Tenth Revision (ICD-10): Paranoid schizophrenia (F20.0), Hebephrenic schizophrenia (F20.1), Catatonic schizophrenia (F20.2), Undifferentiated schizophrenia (F20.3), Post-schizophrenic depression (F20.4), Residual schizophrenia (F20.5), Simple schizophrenia (F20.6), Other schizophrenia (F20.8), Schizoaffective disorder, manic type (F25.0), Schizoaffective disorder, depressive type (F25.1), Schizoaffective disorder, depressive type (F25.2).

### Variables

All data were extracted from a single database, SIA/SUS, which contained the data related to the demographics and clinical variables. The drugs were identified in the database using the code belonging to the SIA/SUS.

Exposure groups were considered according to the SGA used at the cohort entry: clozapine, olanzapine, risperidone, quetiapine and ziprasidone.

The following baseline demographic variables were considered: sex, age at cohort entry, race (reported by the physician), geographic region of residence at study entry, and year of cohort entry (defined as the year of the first provision of SGAs from January 1, 2008, to December 31, 2017). The baseline clinical variables considered were: SGA used at cohort entry, diagnosis according to ICD-10 at cohort entry and mean treatment duration (entire study period) in months.

### Data Source/Measurement

The data on all the patients identified were gathered from the registers of the nationwide database SIA/SUS. The files that make up the database are fragmented by state, year, and month. The records were linked by the National Health Card number for follow-up using deterministic linkage. There was no linkage with another database.

The first date of SGA provision identified in the SIA/SUS database during the period from January 1, 2008, to December 31, 2017, was considered as the cohort entry date. Some patients might already have received SGAs when they entered the cohort.

During the 10-year study period, all patients were followed up until the last patient record in the SIA/SUS or death. Our analysis did not exclude or censor any patient during the period of study.

### Outcomes

The primary outcome was defined as the switch between SGAs. The switch was considered when a patient switched from an oral one SGA to another oral SGA. The following were considered as possible factors associated with SGA switching and that were available in the SIA/SUS: 1) sex, ii) age, iii) year of cohort entry (2008–2017), iv) geographic region of residence; v) diagnosis according to ICD-10 codes, and vi) SGA used at study entry (clozapine, risperidone, quetiapine, ziprasidone and olanzapine).

### Statistical Analysis

Continuous variables were expressed as mean ± standard deviation (SD), and categorical variables as percentages only for descriptive statistics. Pearson’s Chi squared test was performed to analyze correlations between switches and categorical variables. Incidence rates of switches were calculated as cumulative incidence (events/100 patient-years) and compared using the hazard ratio. Absence of switches was estimated using the Kaplan-Meier method. Log rank test was used to assess the significance of differences between absence of switches curves. Cox regression analysis was performed to analyze the association of age, sex, year of cohort entry, geographic region of residence, diagnosis and SGA used at cohort entry. A multivariable Cox proportional-hazards regression model adjusted by all variables was used to examine the hazard ratios of the included factors.

The same procedure was repeated in the sensitivity analysis with patients who entered at cohort in 2009 to exclude eventual cases of patients who were using SGA before 2008. To assess the consistency of the multivariate model, the proportional-hazards assumption on the basis of Schoenfeld residuals was tested. If the model was not proportional, a comparison was made between the investigated factors using the restricted mean survival time.

All tests were two-tailed. Confidence intervals of 95% and a significance level of *p* <  0.05 were used. All statistical analyses were performed using Stata 14.2.

## Results

**FIGURE 1:**
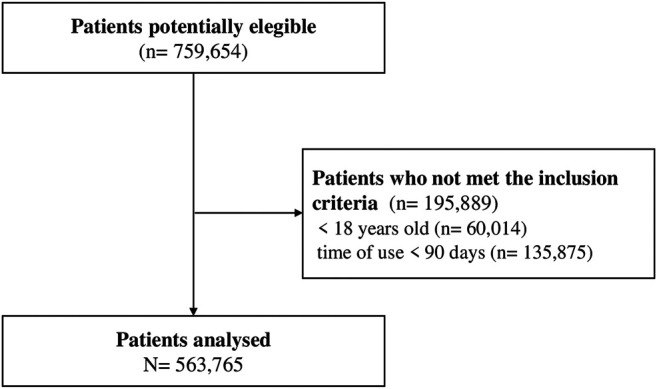
Flow chart of study.

We identified 759,654 patients with schizophrenia or schizoaffective disorder who were on SGA provided by the SUS from 2008 to 2017. From among these patients, 25.8% (n = 195,889) did not met the inclusion criteria due to their age or time of use of SGA. A total of 563,765 patients met the inclusion criteria and were included in the cohort, Figure 1.

Most of the patients were women (51.6%), the mean patient age at study entry was 46.4 (±18.6) years, lived in the south of Brazil (60.3%) and the most commonly diagnosed disorder was paranoid schizophrenia (78.2%). The most commonly used SGA at study entry was olanzapine (31.8%), followed by risperidone (29.2%), quetiapine (28.4%), ziprasidone (5.3%), and clozapine (5.2%). The mean follow-up duration was 27 months (IQR: 12.6–57.8), Table 1.

**TABLE 1:** Demographic and clinic characteristics of study participants according second-generation antipsychotics used.

Variables	Clozapine n = 29,466 (%)	Olanzapine n = 179,486 (%)	Risperidone n = 164,612 (%)	Quetiapine n = 159,989 (%)	Ziprasidone n = 30,212 (%)	Total 563,765
**Sex**						
Male	18,450 (6.7)	99,416 (36.4)	84,188 (30.8)	58,309 (21.3)	12,516 (4.6)	272,879
Female	11,016 (3.8)	80,070 (27.5)	80,424 (27.6)	101,680 (34.9)	17,696 (6.1)	290,886
**Mean age (SD)**	38.91 (±13.2)	42.65 (±16.1)	45.82 (±18.4)	54.42 (±20.6)	40.55 (±12.9)	46.61 (±18.6)
**Age group at cohort entry (years)**						
18–29	8,133 (7.1)	43,640 (37.9)	36,613 (31.8)	20,050 (17.4)	6,709 (5.8)	115,145
30–39	8,610 (7.2)	42,360 (35.7)	33,360 (28.1)	25,998 (21.9)	8,372 (7.0)	118,700
40–49	6,665 (5.9)	38,166 (33.9)	32,372 (28.8)	27,275 (24.3)	7,895 (7.0)	112,373
50–59	3,844 (4.5)	27,716 (32.7)	24,924 (29.5)	23,231 (27.5)	4,894 (5.8)	84,609
60–69	1,454 (3.0)	14,203 (29.2)	14,630 (30.1)	16,602 (34.2)	1,645 (3.4)	48,534
70–79	560 (1.3)	8,447 (19.5)	12,692 (29.3)	21,062 (48.6)	519 (1.2)	43,280
≥ 80	200 (0.5)	4,954 (12.0)	10,021 (24.4)	25,771 (62.7)	178 (0.4)	41,124
**Race**						
White	3,362 (5.3)	17,286 (27.2)	16,130 (25.4)	24,316 (38.2)	2,478 (3.9)	63,572
Black	208 (3.9)	1,898 (36.2)	1,547 (29.5)	1,345 (25.6)	248 (4.7)	5,246
Pardo	1,877 (5.6)	12,099 (36.1)	8,726 (26.0)	9,562 (28.5)	1,278 (3.8)	33,542
Yellow^a^	628 (5.3)	4,617 (39.1)	2,782 (23.5)	3,370 (28.5)	413 (3.5)	11,810
Indigenous	3 (3.9)	16 (21.0)	30 (39.5)	33 (43.4)	5 (6.6)	76
No information	23,388 (5.2)	143,570 (31.9)	135,397 (30.1)	121,374 (27.0)	25,790 (5.7)	449,519
**Year of cohort entry**						
2008	9,167 (7.3)	49,423 (39.2)	40,030 (31.8)	16,746 (13.3)	10,622 (8.4)	125,988
2009	1,816 (3.6)	15,753 (31.8)	16,296 (32.9)	12,053 (24.3)	3,659 (7.4)	49,577
2010	2,049 (4.2)	13,148 (26.7)	16,328 (33.2)	14,294 (29.1)	3,331 (6.8)	49,150
2011	2,480 (4.9)	12,284 (24.6)	18,139 (36.4)	13,991 (28.1)	2,983 (5.9)	49,877
2012	1,700 (4.1)	10,610 (25.8)	13,722 (33.3)	13,214 (32.1)	1,915 (4.6)	41,161
2013	1,903 (4.2)	13,084 (29.0)	12,942 (28.7)	15,432 (34.2)	1,745 (3.9)	45,106
2014	1,730 (3.7)	14,125 (30.0)	12,684 (26.9)	17,170 (36.5)	1,280 (2.7)	46,989
2015	3,060 (5.1)	19,525 (32.8)	13,154 (22.1)	21,954 (36.8)	1,883 (3.2)	59,576
2016	3,944 (6.4)	20,298 (32.7)	13,699 (22.1)	21,936 (35.4)	2,142 (3.4)	62,019
2017	1,617 (4.7)	11,236 (32.7)	7,618 (22.2)	13,199 (38.5)	652 (1.9)	34,322
**Geographic region of residence at study entry**						
South	7,448 (10.7)	22,792 (32.9)	22,139 (32.0)	12,830 (18.5)	3,929 (5.7)	69,138
Northeast	5,060 (5.0)	36,927 (36.7)	29,105 (28.9)	23,354 (23.2)	6,064 (6.0)	100,510
Southeast	13,522 (3.9)	97,447 (28.6)	101,323 (29.8)	110,155 (32.4)	17,834 (5.2)	340,281
North	735 (6.2)	5,079 (43.2)	3,448 (29.3)	2,097 (17.8)	393 (3.3)	11,752
Midwest	2,701 (6.4)	17,241 (40.9)	8,597 (20.4)	11,553 (27.4)	1,992 (4.7)	42,084
**Diagnosis at cohort entry (ICD-10 codes)**						
Paranoid schizophrenia (F20.0)	223,447 (5.3)	143,816 (32.6)	131,072 (29.7)	118,909 (26.9)	23,514 (5.3)	440,758
Hebephrenic schizophrenia (F20.1)	1,681(10.6)	5,329 (33.6)	4,098 (25.9)	3,712 (23.4)	1,014 (6.4)	15,834
Catatonic schizophrenia (F20.2)	207 (8.2)	898 (35.6)	696 (27.6)	583 (23.1)	140 (5.5)	2,524
Undifferentiated schizophrenia (F20.3)	806 (5.7)	4,370 (30.9)	3,687 (26.0)	4,452 (31.4)	840 (5.9)	14,155
Post-schizophrenic depression (F20.4)	74 (2.4)	688 (22.3)	657 (21.3)	1,510 (48.9)	159 (5.1)	3,088
Residual schizophrenia (F20.5)	1,397 (7.6)	6,369 (34.6)	4,824 (26.2)	4,320 (23.4)	1,516 (8.2)	18,426
Simple schizophrenia (F20.6)	212 (3.5)	1,786 (29.5)	2,027 (33.5)	1,693 (28.0)	327 (5.4)	6,045
Other schizophrenia (F20.8)	1,487 (2.5)	14,940 (25.7)	16,727 (28.8)	22,329 (38.5)	2,544 (4.4)	58,027
Schizoaffective disorder, manic type (F25.0)	75 (4.2)	480 (27.2)	293 (16.6)	876 (49.7)	39 (2.2)	1,763
Schizoaffective disorder, depressive type (F25.1)	28 (1.6)	419 (24.8)	308 (18.3)	874 (51.8)	57 (3.4)	1,686
Schizoaffective disorder, mixed type (F25.2)	52 (3.5)	391 (26.8)	223 (15.3)	731 (50.1)	62 (4.2)	1,459
**Median treatment duration (in months) (IQR)**	36.4 (17.3–81.2)	30.9 (14.5–65.7)	26.6 (12.1–56.7)	23.5 (11.6–45.3)	34.6 (15.2–75.0)	27.4 (12.6–57.8)

a Yellow include Asiatic patients and their descendants.

There were sex, age, residence region, and treatment length related differences in the frequency of the SGA used. Clozapine, olanzapine, and risperidone were the most often prescribed SGA among men in the entry of the study. The proportion of quetiapine and ziprasidone use among women was higher than that in men.

Quetiapine was more frequently used by elderly individuals aged higher than 60 years, and the frequency of use increased progressively with advancing age (60–69 years: 34.2%; 70–79 years: 48.6%; ≥80 years: 62.7%).

The frequency of SGA use differed among the various regions of Brazil. In the southeast region, quetiapine (32.4%) and risperidone (29.8%) were the most used, while in the northeast region, the most commonly used SGAs were olanzapine and risperidone. The median treatment duration varied from approximately 23 (IQR: 11.6–45.3) months for quetiapine to 36 (17.3–81.2) months for clozapine.

Almost all (99.9%) switched the SGA at least once during follow-up. Female sex, advanced age (>70 years old), residence in the northeast region, study entry from 2009, and a diagnosis of schizoaffective disorders were associated with an increased risk of switching (*p* < 0.001 for each variable, Table 2). Compared to the adjusted hazard ratio for clozapine users, the corresponding ratios for risperidone, ziprasidone, quetiapine and olanzapine users were 1.59 (95% CI, 1.57–1.61), 1.41 (95% CI, 1.39–1.44), 1.25 (95% CI, 1.23–1.26), and 1.11 (95% CI, 1.10–1.12).

**TABLE 2:** Factors associated with second generation antipsychotics switch.

Variables	N	Incidence of switches^a^	Hazard Ratio 95% CI not adjusted	*p* value	Hazard Ratio 95% CI Adjusted^b^	*p* value	Hazard Ratio 95% CI Sensitivity analysis^c^	*p* value
**Sex**								
Males	272,879	0.48	1.00		1.00		1.00	
Females	290,886	0.53	1.10 (1.09–1.11)	<0.001	1.05 (1.05–1.06)	<0.001	1.05 (1.04–1.06)	<0.001
**Age group at study entry (years)**								
18–29	115,145	0.50	1.00		1.00		1.00	
30–39	118,700	0.48	0.95 (0.94–0.96)	<0.001	0.94 (0.93–0.94)	<0.001	0.93 (0.92–0.94)	<0.001
40–49	112,373	0.46	0.92 (0.91–0.93)	<0.001	0.90 (0.89–0.90)	<0.001	0.89 (0.88–0.90)	<0.001
50–59	84,609	0.48	0.97 (0.96–0.98)	<0.001	0.89 (0.88–0.90)	<0.001	0.87 (0.86–0.88)	<0.001
60–69	48,534	0.52	1.04 (1.03–1.06)	<0.001	0.92 (0.91–0.93)	<0.001	0.89 (0.88–0.90)	<0.001
70–79	43,280	0.59	1.18 (1.17–1.19)	<0.001	1.03 (1.01–1.04)	<0.001	0.98 (0.97–0.99)	0.021
≥ 80	41,124	0.68	1.36 (1.34–1.38)	<0.001	1.14 (1.12–1.15)	<0.001	1.09 (1.08–1.11)	<0.001
**Year of cohort entry**								
2008	125,988	0.33	1.00		1.00			
2009	49,577	0.41	1.36 (1.34–1.37)	<0.001	1.32 (1.31–1.34)	<0.001	1.00	
2010	49,150	0.44	1.51 (1.49–1.53)	<0.001	1.45 (1.43–1.46)	<0.001	1.17 (1.15–1.18)	<0.001
2011	49,877	0.45	1.58 (1.57–1.60)	<0.001	1.56 (1.54–1.57)	<0.001	1.32 (1.31–1.34)	<0.001
2012	41,161	0.50	1.79 (1.77–1.81)	<0.001	1.74 (1.72–1.76)	<0.001	1.52 (1.50–1.54)	<0.001
2013	45,106	0.54	1.97 (1.95–1.99)	<0.001	1.91 (1.89–1.93)	<0.001	1.72 (1.70–1.75)	<0.001
2014	46,989	0.65	2.41 (2.38–2.43)	<0.001	2.35 (2.32–2.38)	<0.001	2.20 (2.17–2.23)	<0.001
2015	59,576	0.74	2.77 (2.74–2.80)	<0.001	2.80 (2.77–2.83)	<0.001	2.69 (2.66–2.73)	<0.001
2016	62,019	0.95	3.76 (3.72–3.80)	<0.001	3.85 (3.81–3.89)	<0.001	3.84 (3.79–3.90)	<0.001
2017	34,322	1.86	9.59 (9.46–9.72)	<0.001	9.50 (9.37–9.63)	<0.001	9.81 (9.66–9.97)	<0.001
**Geographic region of residence at study entry**								
South	69,138	0.47	1.00		1.00		1.00	
Northeast	100,510	0.60	1.29 (1.28–1.30)	<0.001	1.31 (1.30–1.33)	<0.001	1.37 (1.36–1.39)	<0.001
Southeast	340,281	0.48	1.06 (1.05–1.07)	<0.001	1.12 (1.11–1.13)	<0.001	1.18 (1.17–1.20)	<0.001
North	11,752	0.53	1.16 (1.14–1.18)	< 0.001	1.26 (1.23–1.28)	<0.001	1.33 (1.30–1.36)	<0.001
Midwest	42,084	0.50	1.09 (1.07–1.10)	<0.001	1.12 (1.11–1.13)	<0.001	1.18 (1.16–1.20)	<0.001
**Diagnosis at study entry (ICD-10 codes)**								
Paranoid schizophrenia (F20.0)	440,758	0.49	1.00		1.00		1.00	
Hebephrenic schizophrenia (F20.1)	15,834	0.47	0.94 (0.92–0.95)	<0.001	0.93 (0.91–0.94)	<0.001	0.92 (0.91–0.94)	<0.001
Catatonic schizophrenia (F20.2)	2,524	0.48	0.97 (0.93–1.00)	0.13	0.99 (0.95–1.03)	0.71	0.97 (0.93–1.02)	0.35
Undifferentiated schizophrenia (F20.3)	14,155	0.52	1.05 (1.03–1.07)	<0.001	0.98 (0.96–0.99)	0.02	0.98 (0.96–0.99)	0.03
Post-schizophrenic depression (F20.4)	3,088	0.50	1.00 (0.97–1.04)	0.64	0.95 (0.92–0.98)	0.01	0.97 (0.94–1.01)	0.24
Residual schizophrenia (F20.5)	18,426	0.44	0.89 (0.88–0.90)	<0.001	0.90 (0.89–0.92)	<0.001	0.90 (0.88–0.91)	<0.001
Simple schizophrenia (F20.6)	6,045	0.54	1.08 (1.05–1.10)	<0.001	0.99 (0.96–1.01)	0.56	0.99 (0.96–1.02)	0.62
Other schizophrenia (F20.8)	58,027	0.55	1.10 (1.09–1.11)	<0.001	1.01 (1.00–1.02)	0.001	1.02 (1.01–1.03)	<0.001
Schizoaffective disorder, manic type (F25.0)	1,763	1.13	2.26 (2.15–2.36)	<0.001	1.09 (1.04–1.14)	<0.001	1.11 (1.05–1.16)	<0.001
Schizoaffective disorder, depressive type (F25.1)	1,686	1.10	2.19 (2.09–2.30)	<0.001	1.10 (1.05–1.16)	<0.001	1.13 (1.07–1.18)	<0.001
Schizoaffective disorder, mixed type (F25.2)	1,459	1.08	2.13 (2.02–2.24)	<0.001	1.11 (1.05–1.17)	<0.001	1.13 (1.07–1.19)	<0.001
**SGA used at study entry**								
Clozapine	29,466	0.37	1.00		1.00		1.00	
Olanzapine	179,486	0.43	1.15 (1.14–1.17)	<0.001	1.11 (1.10–1.12)	<0.001	1.08 (1.06–1.10)	<0.001
Risperidone	164,612	0.58	1.58 (1.56–1.60)	<0.001	1.59 (1.57–1.61)	<0.001	1.52 (1.50–1.54)	<0.001
Quetiapine	159,989	0.57	1.56 (1.54–1.58)	<0.001	1.25 (1.23–1.26)	<0.001	1.17 (1.15–1.19)	<0.001
Ziprasidone	30,212	0.47	1.29 (1.27–1.31)	<0.001	1.41 (1.39–1.44)	<0.001	1.36 (1.33–1.39)	<0.001

a Incidence/100 patients-years.

b Adjusted by all variables.

c Adjusted by all variables without 2008 year.

The incidence of switching was 58.2/100 person-years for risperidone users, followed by 57.6/100 person-year for quetiapine users, 49.8/100 person-years for ziprasidone users, 43.5/100 person-years for olanzapine users and 37.6/100 person-years for clozapine users. Risperidone was associated with the highest risk of switching, while the corresponding risk associated with clozapine was the lowest.

**FIGURE 2:**
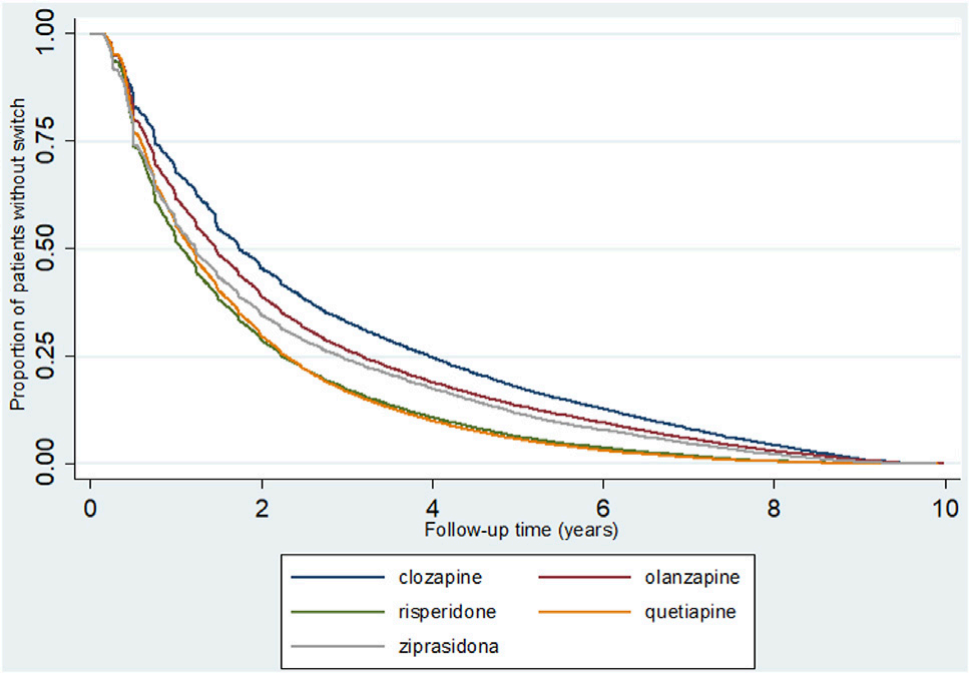
Maintenance of the use of the same antipsychotic (without switch) during the study period, Kaplan-Meier curve.

Figure 2 shows the maintenance of the use of the same SGA during the follow-up period, with time without switching. Patients who used clozapine remained free from switching for a longer duration. In the sensitivity analysis (excluding patients from 2008), we did not identify any important differences in the findings. The result of the tests of proportional-hazards assumption was significant (*p* < 0.05). When repeating the analysis using the restricted mean survival time, the factors associated with switch of SGA were the same.

## Discussion

### Main Findings

Increased risk of SGA switching was associated with female sex, advanced age, a diagnosis of schizoaffective disorder, and type of SGA used at study entry. Risperidone users were more likely to switch to another SGA, while clozapine users were less likely to switch than other users.

### Comparison with Previous Studies

Switching antipsychotics is a common clinical practice in schizophrenia management (Sernyak et al., 2005; Kreyenbuhl et al., 2007; Nyhuis et al., 2010). Our findings confirmed this, and we found that the switches happened at least once in 10 years. The SGAs are similar in terms of effectiveness, except clozapine, but show significant differences in the tolerability profile and adverse reactions, which may explain the switch (Siskind et al., 2016; Preda and Bora, 2019).

Women were more likely to switch SGAs than were men. This finding is consistent with that in other studies (Barbui et al., 2005; Nyhuis et al., 2010) and could be attributed to sex differences and to the fact that women use more health services than do men and, in turn, could detect a suboptimal treatment response or serious adverse event early (Lindamer et al., 2003). There are sex differences in the tolerability and maintenance regimens of antipsychotics in patients with schizophrenia (Seeman, 2004; Barbui et al., 2005; Leotsakou et al., 2008). Women are less tolerant than men to antipsychotics (Barbui et al., 2005). Furthermore, women tend to report adverse effects, such as extrapyramidal and anticholinergic reactions, hyperprolactinemia, and weight gain, more frequently, whereas men report more sexual problems (Barbui et al., 2005; Schwartz et al., 2015; Alberich et al., 2019). Women and men could not be considered a homogenous group in the use of antipsychotics (Seeman, 2004).

Advanced age was another factor associated with switching, which conflicts with other findings, whose age group of switchers was between 42 and 55 years (Kreyenbuhl et al., 2007; Nyhuis et al., 2010). This discrepancy can be attributed to the size of our sample (n = 563,765). Our findings come from a large and representative sample (whose results were confirmed by a sensitivity analysis adjusted for several confounders), which differ from studies with smaller samples that did not find an association with advanced age. Another study showed that advanced age reduced the likelihood of switching overall which was attributed to some hypotheses like greater clinical stability or low clinician expectations of potential benefit of antipsychotics switch in older people (Sernyak et al., 2005). But more researches are needed to elucidate it.

Residents of the northeast region of Brazil were more likely to switch SGAs, which may reflect of socioeconomic factors. The northeast region is one of the poorest regions in Brazil, with high rates of unemployment, illiteracy, and poor basic sanitation. It succeeds the southeastern part in the number of schizophrenia cases in Brazil and is considered a vulnerable region in terms of the development of this disease, and treatment gaps and non-adherence (Dornelas et al., 2019). Although the country offers universal coverage, there is a shortage of doctors and specialized health professionals in certain regions, (Oliveira et al., 2017), as well as a lack of availability of medicines, with the northeast and northern regions being the most affected. Inadequate availability of psychiatrists and nurses in mental health facilities are considered a significant predictor of treatment gap (Lora et al., 2012).

The subtypes of schizophrenia are generally not studied separately and studies involving patients with schizophrenia also include patients with schizoaffective disorder (Brazil, 2014a; Leposavic et al., 2015; Mattila et al., 2015). Although it did not seem predictor of treatment response (Mattila et al., 2015), a diagnosis of schizoaffective disorders, compared to that of paranoid schizophrenia, was likely to associated with SGA switching, as found in our study. Although relevant data are scarce and evidences pertaining to schizophrenia also includes evidence pertaining to schizoaffective disorders, the effectiveness and tolerability profile of SGAs may differ across these conditions (Murru et al., 2016).

The use of risperidone was associated with a higher risk to switching in our cohort. Despite widespread use, low price, and no clear differences in effectiveness compared to those of other SGAs risperidone produces more movement disorders and prolactin increase than do other SGAs (Komossa et al., 2011). In a study conducted among schizophrenia outpatients, in 37 countries (n = 17,000 patients) in which the switches from risperidone to olanzapine and vice versa were compared, patients who switched from risperidone to olanzapine exhibited more favorable outcomes and remained on the medication longer (Hong et al., 2012). In another systematic review (15 studies, n = 7760 patients), a comparison between SGAs showed that risperidone was slightly less acceptable than olanzapine (Komossa et al., 2011).

Patients using clozapine switched less than did patients using other SGAs. Meta-analyses have shown that clozapine is more effective than other SGAs, being superior for positive symptoms in the short and long term (Asenjo Lobos et al., 2010; Siskind et al., 2016). Regarding negative symptoms, clozapine was superior only in the short term (Siskind et al., 2016). A cohort study conducted in Sweden among patients (n = 26,046) diagnosed with schizophrenia and using antipsychotics showed that clozapine users were most likely to refill prescriptions and had lower rates of re-hospitalization and death by suicide (Ringback Weitoft et al., 2014). Other studies also highlighted that the adherence rate was significantly higher in patients treated with clozapine (Cooper et al., 2007; Ascher-Svanum et al., 2008). These significant clinical benefits may have contributed to clozapine users switching less than other SGA users in our cohort.

However, clozapine is used less frequently and later in treatment. Studies have shown that there are long delays in starting treatment with clozapine in resistant patients (Wheeler, 2008; Howes et al., 2012; Alessi-Severini et al., 2013). Guidelines for schizophrenia treatment do not recommend clozapine as the first option (Brazil, 2013; Kuipers, 2014; Remington et al., 2017; Preda and Bora, 2019). It is offered only to patients who have not responded adequately to adequate doses of at least two different antipsychotics. This is probably due to the risk of inducing agranulocytosis (almost 1%), which can be fatal, and the need for closer monitoring (Preda and Bora, 2019; Van Zuuren, 2019). Other concerns include the risk of cardiovascular diseases. To ensure a balance between effectiveness and safety and considering real-world information about the use of clozapine in outpatients, guidelines could be reviewed and different recommendations could be made.

### Strengths and Limitations of the Study

This is the first national cohort study that assessed the factors associated with switching of SGAs dispensed by the Brazilian public sector over 10 years. The use of a nationwide pharmaceutical database allowed us to investigate a large quantity of data on the use of antipsychotics in real-life and to obtain a representative and very large patient population with schizophrenia. Our clear and comprehensive methodology led to robust findings to the public health which could direct future researches.

There are some limitations of this study. First, despite the reliability of the data, the diagnoses were not validated. Second, SIA/SUS is an administrative database that does not consider dispensation from private pharmacies, which could lead to an underestimation of the use of these antipsychotics. Nevertheless, APAC/SIA covers more than 70% of the Brazilian population (more than 148 million inhabitants). Third, as an administrative database, it is not intended for research purposes and may have system data feed errors. However, we carefully checked data inconsistencies to avoid this type of bias and excluded patients who received antipsychotics only once or for less than 90 days. Fourth, detailed clinical data, comorbities, use of concomitant drugs, as well as the reasons that determined the switches are unavailable because the original purpose of this database is to register the consumption, the charge, and the payment of dispensing high-cost medicines.

## Conclusion

This nationwide cohort study elucidated the factors associated with SGAs switching in a real-world setting. The choice of the prescribed antipsychotic can determine the trend for switching and affect the long and short-term outcomes for patients with life-long disorders. The findings of our study have implications for future research.

## Data Availability

The raw data supporting the conclusions of this article will be made available by the authors, without undue reservation.
